# Exploration of umbilical hernia incidence and etiology in 753 cases of single-incision laparoscopic surgery: a retrospective analysis

**DOI:** 10.1186/s12893-025-02958-x

**Published:** 2025-05-22

**Authors:** Peng Chen, Jingyi Jiao, Huimin Xue, Xiaojun Zhu, Xiaojuan Wang, Peng Wang

**Affiliations:** 1 Nantong University Medical School, Nantong, 226001 China; 2 Department of Hepatobiliary and Pancreatic Surgery, Affiliated Hospital of Nantong University, No.20 Xisi Road, Nantong, Jiangsu 226001 China

**Keywords:** Closing method, Incisional hernia, Surgical procedure, Single incision laparoscopic surgery, Umbilical hernia

## Abstract

**Purpose:**

The rising popularity of Single Incision Laparoscopic Surgery (SILS) brings concerns regarding a higher incidence of postoperative incisional hernias due to the enlarged umbilical incision compared to conventional laparoscopy. This study aims to explore the occurrence of incisional hernias following single-port laparoscopic surgery and identify associated risk factors.

**Methods:**

The patient cohort included individuals who underwent cholecystectomy and inguinal hernia repair procedures using the SILS technique. Follow-up assessments were conducted via written correspondence, telephone interviews, and clinical examinations. Univariate and multivariate analyses were employed to investigate the impact of demographic variables and surgical parameters, including age, gender, BMI, ASA score, operative duration, pre-existing umbilical hernia, and the occurrence of postoperative incisional hernia.

**Results:**

A total of 753 patients completed follow-up, with a mean duration of 60.2 months and variance: 51.26. Among them, 342 (45.4%) underwent cholecystectomy, while 411 (54.6%) underwent inguinal hernia repair. The study cohort comprised 405 women (53.8%) and 348 men (46.2%), with a mean age of 40 years (range 10–83 years) at the time of surgery. Only one patient (0.13%) required conversion to conventional laparoscopy for surgical access. Intraoperative complications occurred in 0.1% of cases, while postoperative complications occurred in 1.6%. Incisional hernias developed in 10 patients (1.3%), with a notably higher incidence of 5.9% among obese patients than normoweight patients. Additionally, 23.1% of patients with pre-existing umbilical hernias experienced incisional hernia during the follow-up period. Multivariate analyses revealed that obesity(OR: 18.56, Cl:5.76–86.42, p value:0.003), pre-existing umbilical hernia(OR:16.32,Cl:4.26–61.68, p value:0,002), diabetes(OR:2.42, Cl:1.86–20.42, p value:0.496), and hypertension(OR:1.96, Cl:0.72–12.64,p value:0.924) were significantly associated with incisional hernia incidence. However, gender, age, type of surgery (inguinal hernia repair vs. cholecystectomy), presence of acute inflammation, and duration of surgery did not show statistically significant associations with incisional hernia occurrence.

**Conclusion:**

Detecting incisional hernias necessitates an extended follow-up period. In the univariate analysis, obesity and pre-existing umbilical hernias were linked to an elevated risk of this complication. Following meticulous patient selection, Single Incision Laparoscopic Surgery (SILS) presents a secure method for performing cholecystectomy and inguinal hernia repair.

## Introduction

Minimally invasive laparoscopic surgery has been a hot topic in recent years, and has gained popularity among many patients and surgeons for its less invasive nature, fewer complications, and good cosmetic benefits [1–3]. Most appendectomies and inguinal hernia repairs are now done by laparoscopic surgery [1, 2, 4, 5], and with the promotion and popularity of the SILS procedure, it has been found to further reduce tissue damage and improve cosmetic advantages [6, 7]. Although conventional laparoscopic surgery is still the gold standard for the treatment of gallbladder stones and inguinal hernias, SILS is gaining more and more popularity among patients, and its ability to be virtually scarless by virtue of its natural orifice [8], the navel, as a surgical entry point is undoubtedly an excellent choice for patients working in an industry with very high cosmetic requirements [3]. However, in recent years, the issue of increased incidence of umbilical hernia associated with single-incision laparoscopic surgery (SILS) has drawn increasing attention and discussion among surgeons. Jordi Comajuncosas et al. [9] reported an alarmingly high umbilical hernia incidence of 25.9% following single-incision laparoscopic surgery (SILS), highlighting associated complications including increased infection risk and other drawbacks. Conversely, a subsequent systematic review by M Rhodes et al. documented an overall incidence of 3–10% for this complication [10]. However, in our clinical practice involving over 1,000 SILS procedures, we observed an extremely low recurrence rate with no umbilical hernia cases identified during routine follow-up and outpatient evaluations. Therefore, we believe it is critical to share our detailed technical nuances and propose surgical modification strategies to optimize outcomes for both patients and surgeons.

SILS requires multiple trocars to be placed in the umbilical incision, which undoubtedly requires a larger fascial incision [11]. Therefore a growing number of reports in the literature have found an increased incidence of incisional hernia after SILS [12]. If this incidence is indeed increased, the cosmetic advantages of SILS over the serious complications of incisional hernia [13]: bowel obstruction and ischaemic necrosis, will be nowhere to be seen. At the same time, follow-up bias, or the fact that some umbilical hernias are small and not obvious, can lead to large discrepancies in follow-up results [14].

In this experimental team, the lead surgeon has been performing SILS for a long time, yet postoperative umbilical hernias have rarely occurred during postoperative patient follow-up and clinical observations. With this in mind, we collected clinical data and long-term follow-up to further understand the incidence of postoperative umbilical hernia after SILS and to analyse the factors that contribute to this complication. Finally, we share the details of our team’s management of the SILS procedure so that this undesirable complication can be effectively avoided.

## Materials and methods

Between March 2019 and March 2024, all patients included in this study underwent surgery at a hospital in China (Affiliated Hospital of Nantong University). Permission for follow-up was obtained from the Ethics Committee of Affiliated Hospital of Nantong University (IRB approval). All patients were fully informed about single- and multi-port laparoscopic surgery prior to surgery. They were all informed about the advantages and limitations of both procedures. The decision to perform SILS surgery for all patients was made by the attending surgeon based on the assessment of the patient’s condition. A comprehensive preoperative assessment was performed to evaluate cardiac function, blood pressure, mental status, and cooperative ability to determine surgical tolerance. Additionally, single-incision laparoscopic surgery (SILS) has relatively low indication requirements. In general, patients without significant organic diseases (e.g., cardiopulmonary or renal impairment), who can tolerate approximately 30-minute surgical duration, demonstrate normal mental status, and pass the preoperative evaluation by anesthesiologists are considered to have adequate physical status for SILS. Demographic variables, surgical parameters and prognostic data were obtained and evaluated from a prospective database and retrospectively analysed for factors such as gender, years, BMI, American Society of Anaesthesiologists (ASA) scores, history of previous abdominal surgeries, diagnosis, duration of surgery, intraoperative transfers(converted to multi-port laparoscopic surgery or conventional open surgery intraoperatively ) as well as postoperative complications, and underlying diseases of the patients. Operative time was defined as the time from skin incision to wound dressing. Length of stay was calculated as the time of surgery (counted as day 0) to discharge. All SILS procedures in this study were performed by the same experienced surgeon [15]. During follow-up, patients underwent a comprehensive evaluation of their single-incision laparoscopic surgery (SILS) experience, assessing satisfaction and willingness to undergo the procedure again or recommend it to others. Among 753 evaluable patients, 741 (98.4%) reported satisfaction and expressed willingness to recommend SILS.

### Surgical procedure

All the cases included in the study used the same intra-abdominal approach and fascial closure technique [16]. Prophylactic antibiotics, including standard cephalosporins and roxithromycin, were administered to all patients, with regimens tailored to individual allergy status.

### Preoperative umbilical incision and puncture device placement

**Fig. 1:**
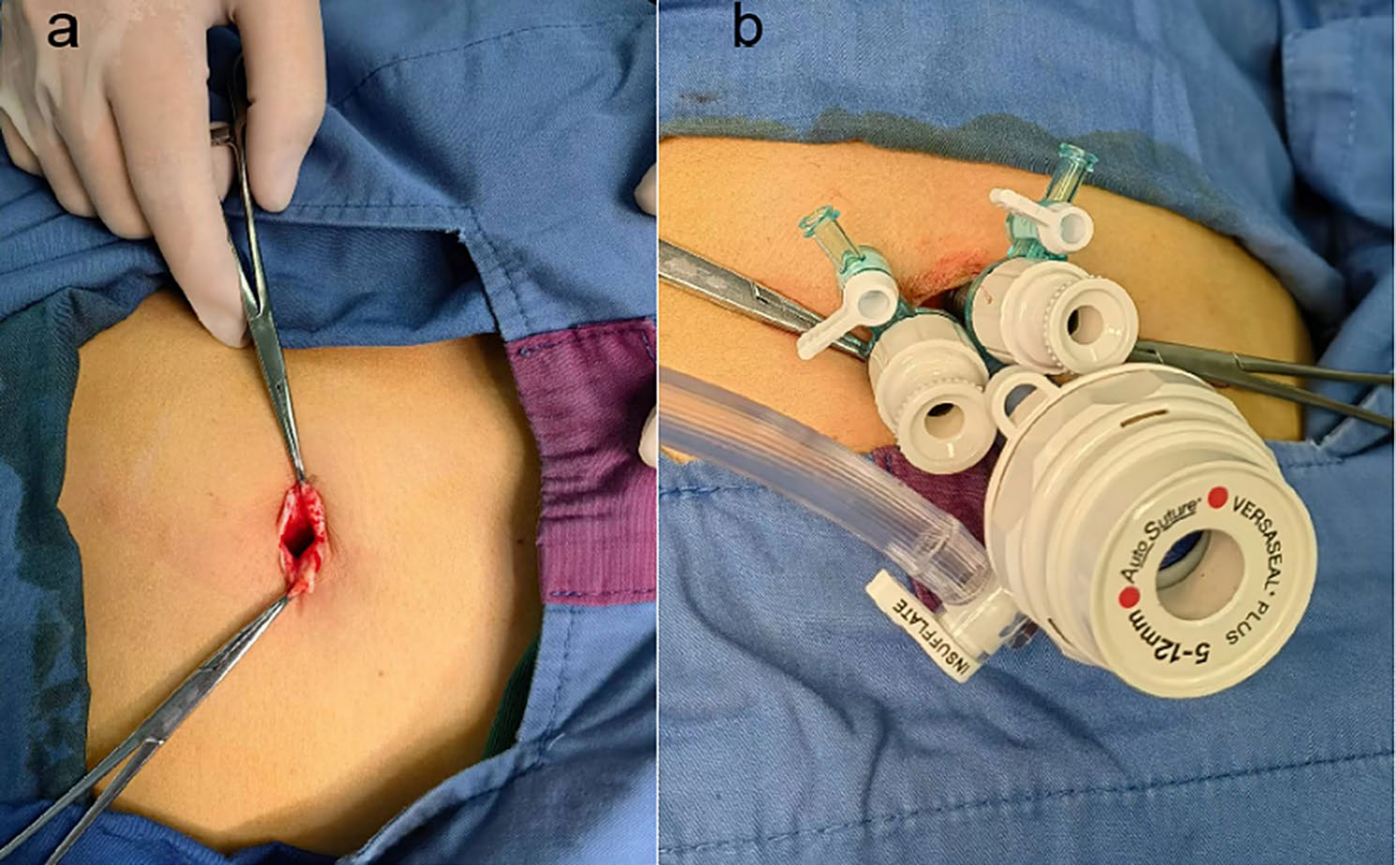
Longitudinal incision of the navel and placement of the piercing device

The umbilical approach was selected, the routine disinfection area was used, the navel was soaked with iodophor, the alcohol cotton ball was picked up by Alice forceps and placed into the navel for disinfection 2–3 times, and the dirt was removed. Longitudinally cut the navel, completely free the umbilical ring, vascular forceps grab the inside of the flap, open to both sides. The skin knife continues to cut down the fascia layer to the peritoneum. As shown in Fig. 1, attention should be paid to patients with a history of abdominal endoscopic surgery to avoid damage to the intestinal duct [17]. This step should be performed with caution.

**Fig. 2:**
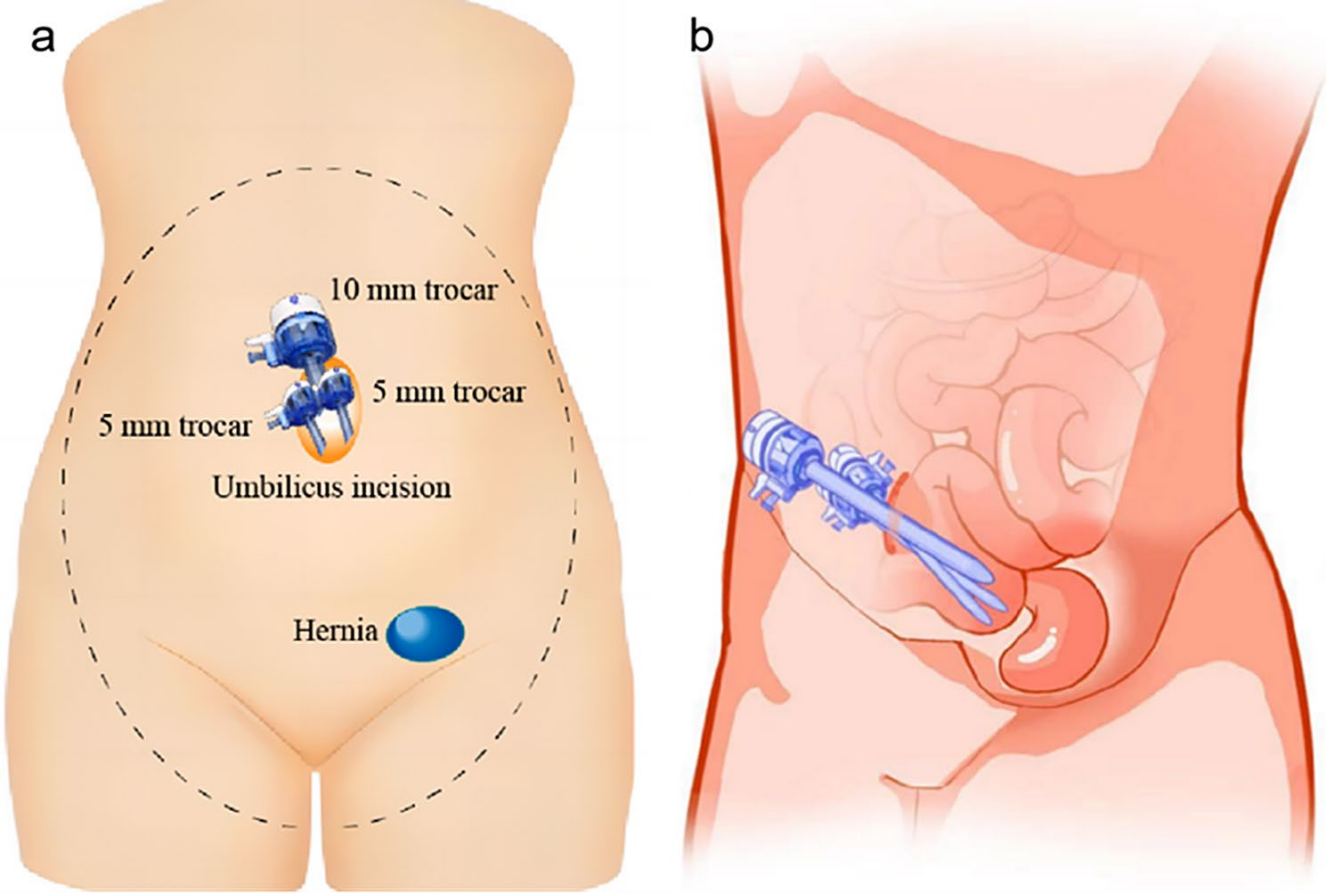
Layout of the trocar

As shown in Fig. 2, the 12 mm puncture device was inserted, the pneumoperitoneum was placed over the large puncture device, and two 5 mm puncture devices were further inserted into the fascia layer as required. The depth of the three piercings can be adjusted according to the needs during the operation, to obtain the space for mechanical operation.

### Closure of the fascial layer after surgery

**Fig. 3:**
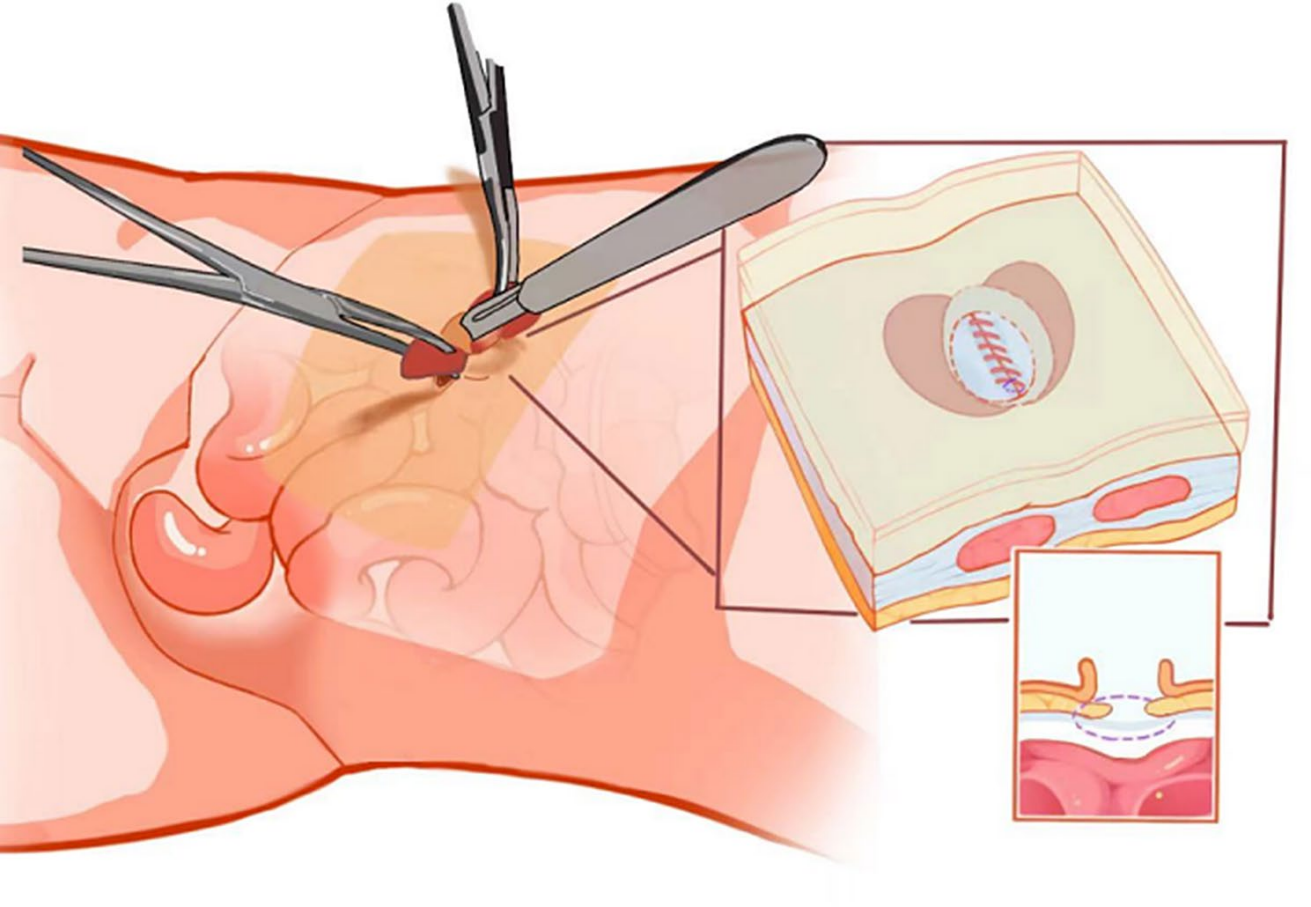
The 1 − 0 barb line closes the fascia layer

We closed the pneumoperitoneum, removed the puncture apparatus, and surgical specimens were removed [18]. When the fascia layer is closed, the function of the free umbilical ring is shown before surgery. It is recommended to use 1 − 0 barb wire for continuous suture because of its uniform force, better blood supply at the edge of the incision, and not easy early fracture of the fascia layer [19]. The distance between the needle points in and out is moderate and should not be too large. This will lose the space of the umbilical socket, which is not conducive to returning the navel to the umbilical socket later, or even cause the navel to protrude [20, 21]. During the suture process, fingers can be used to explore the incision to ensure that the omentum and bowel are not damaged. After the incision in the fascia layer is completely closed, the fingers are also used to explore whether there is any gap, and two continuous edge stitching can be performed to strengthen the incision [22]. Similarly, the needle spacing in and out should not be too large. The process steps are shown in Fig. 3.

### Methods of navel repair

**Fig. 4:**
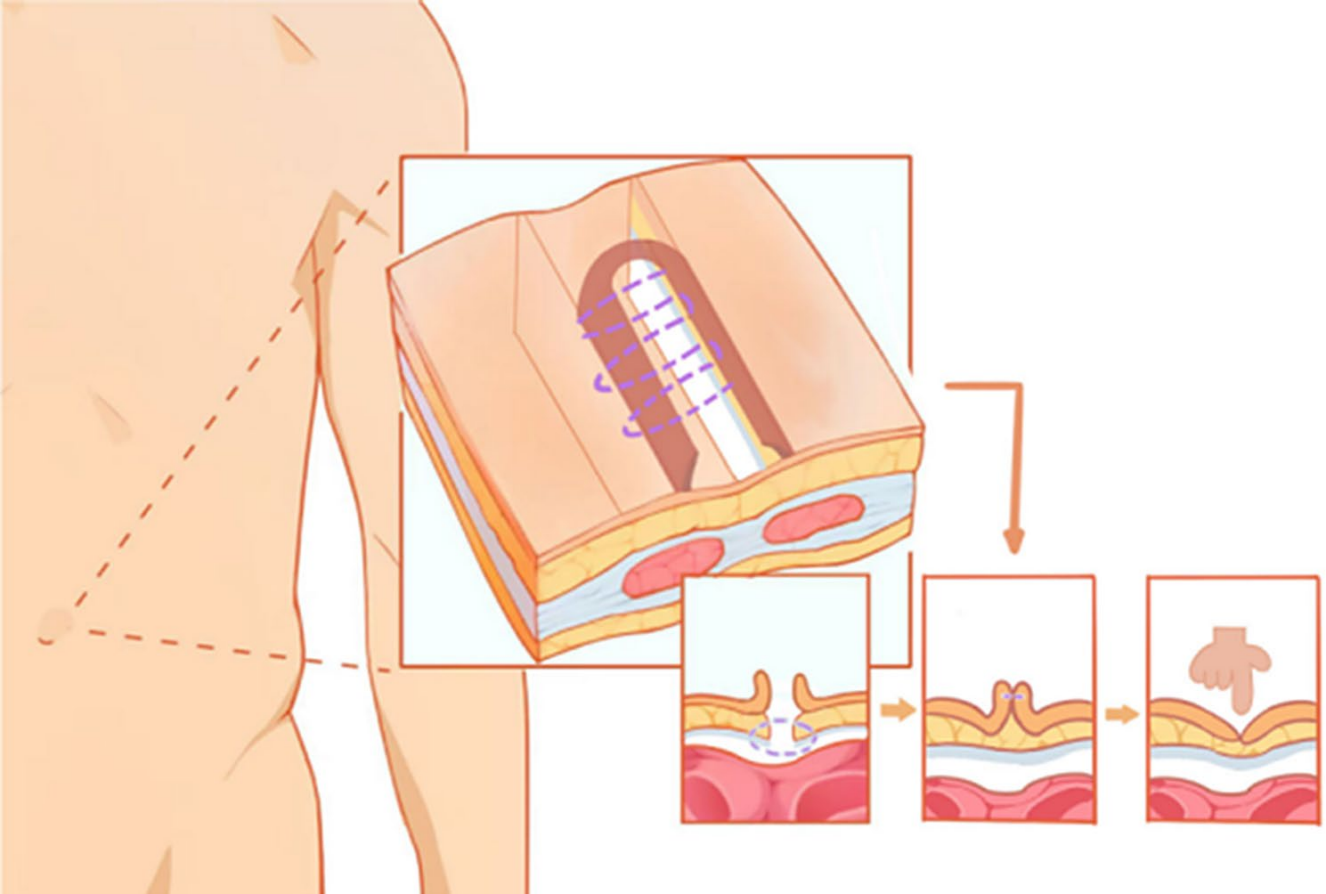
Suture the fascia layer, retract and maintain the umbilical hollow

After the fascia layer is closed, the vascular clamp can be loosened and the two sides of the navel can be turned into the umbilical fossa [23]. If insufficient space is found in the umbilical fossa, scissors can be used to properly loosen the subcutaneous fat layer. We believe that there is no need to close the subcutaneous fat layer. It is recommended to use two 5 − 0 collagen sutures to close the navel, which is quick to absorb and has a good beauty effect. Take the middle point of one side of the navel skin margin as the needle entry point, first out of the needle, and then from the lowest fascia layer into the needle and out of the bottom of the anchor, and then to the other side of the navel skin margin into the needle and out of the needle, do not tie, cut the thread, use two vascular forceps to fix the suture on both sides of the umbilical cord. Another collagen thread was used for continuous intradermal suture from the incised edge of the navel, as depicted in Fig. 4. The two edges of the skin were aligned as far as possible, and the knots of the initial point and the end point were buried in the incision [24]. Push the navel back to the umbilical fossa, use vascular forceps to hold the gauze to absorb subcutaneous congestion, tie the first collagen suture, can fit the midpoint of the navel and the lowest part of the umbilical fossa, and maximize the restoration of the patient’s preoperative navel [25]. Local injection of ropivacaine (stock solution can be used) can significantly reduce postoperative umbilical pain. Wipe the blood with wet gauze and insert 1–2 alcohol cotton balls into the navel.

### Postoperative management of incisions

**Fig. 5:**
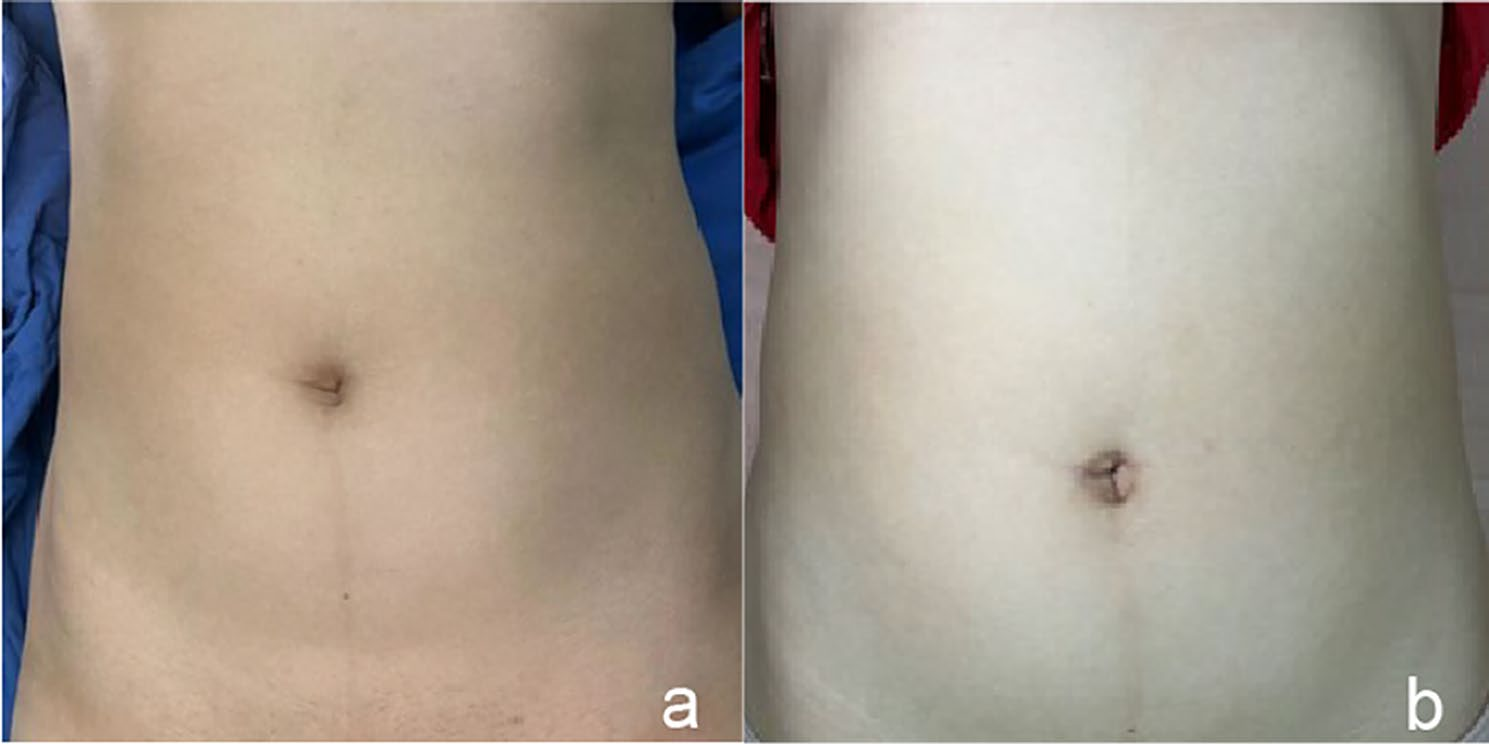
Preoperative(**a**) and postoperative(**b**) comparison of the umbilical cord

There will be some edema in the navel after the operation, an incision dressing change should be performed on the first day after the operation, and the incision should be observed to see if there is any effusion, infection or suture collapse [20]. Generally, the skin of the navel has been fitted to the navel socket on the first day after the operation. If the fitting is not complete, pressure dressing with an alcohol cotton ball can be continued, and the dressing change can be done until the scab is formed the next day. As shown in Fig. 5 below, a is the preoperative photo and b is the postoperative photo.

### Follow-up

**Fig. 6:**
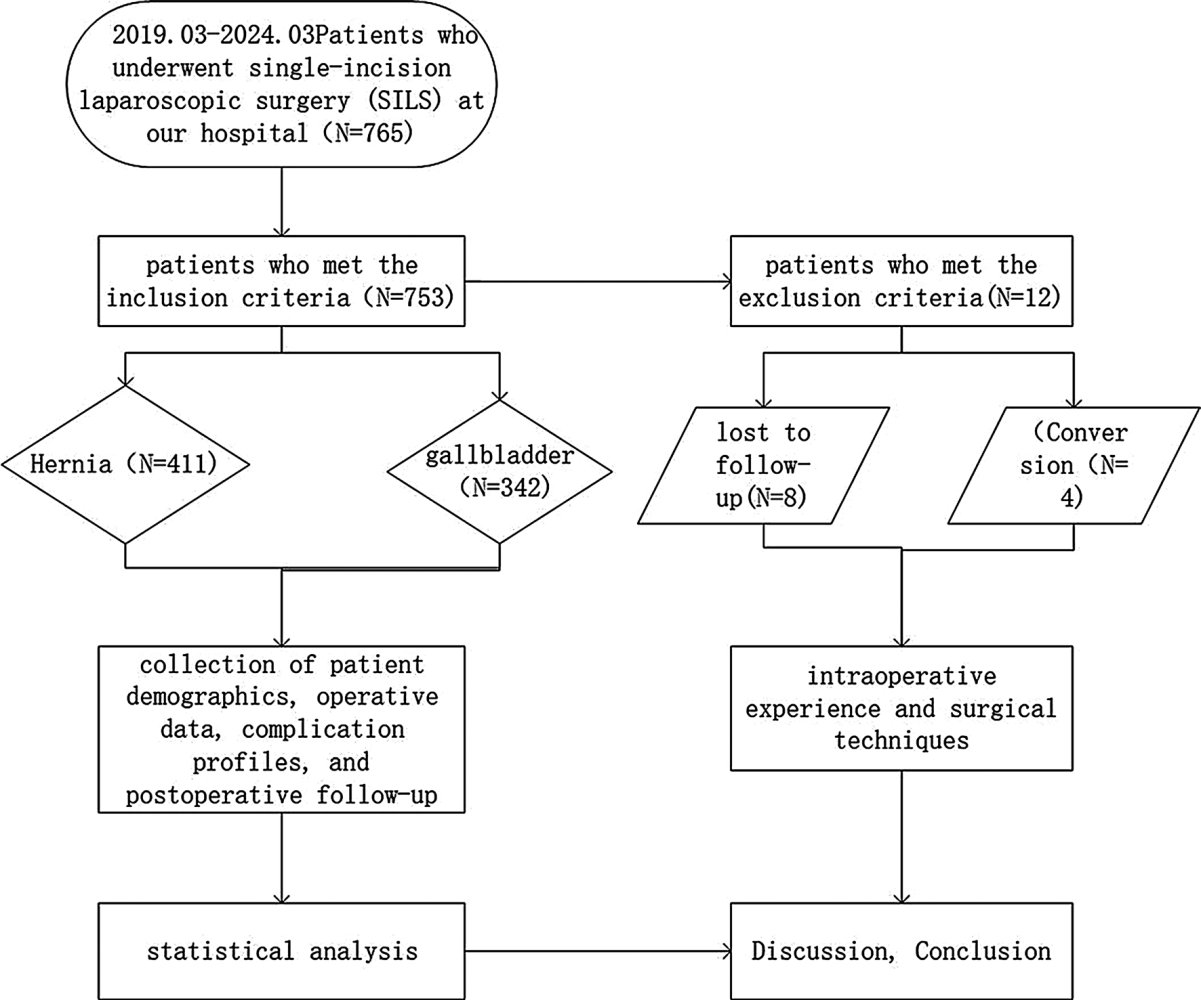
Flowchart of Patient Inclusion and Exclusion Criteria

A total of 753 patients were screened and categorized according to the inclusion criteria, and a systematic analysis was performed following the predefined flow diagram, as illustrated in Fig. 6.Patients were followed up by telephone, email correspondence and clinical examination. Patients were contacted in the following ways: firstly by telephone, and at the same time the questionnaire was distributed by mail. If three attempts to contact the patient failed and there was no email response, the patient was marked as lost. The main questions asked over the phone or by mail were aimed at detecting the occurrence of umbilical hernia symptoms: (1) whether the patient felt any pain or discomfort in the abdomen; (2) whether the umbilicus was protruding or swollen; (3) whether he/she had undergone surgery related to the puncture site, such as a hernia repair, during this period of time; (4) if he/she was experiencing any of the above symptoms, he/she was invited to come to the clinic for a medical check-up and an ultrasound review. A positive ultrasound will confirm that the patient has an umbilical hernia and thus confirm the postoperative complications of SILS [26].

We consider that no umbilical hernia has occurred if the patient perceives no abnormal pain or bulging at the incision site [26]. We will exclude a patient from the follow-up list if an intraoperative transit occurs. The clinical outcomes and benefits of the SILS procedure were examined by scoring the umbilical scar and pain on a scale of 1–10, with the former indicating very satisfactory and virtually painless and the latter indicating very unsatisfactory and very painful.

### Statistical analysis

Statistical analyses were performed using IBM SPSS Statistics (version 25.0). (Almonk, NY: IBM Corp). Continuous variables were tested using independent samples t-tests, and Mann-Whitney U-tests were used for unequal variances. Categorical variables were tested using the chi-square test or Fisher’s exact test. For univariate analyses, the threshold for continuous variables was set as the rounded arithmetic mean of the two corresponding means for that particular variable in patients with incisional hernia versus those without incisional hernia [27]. Binary logistic regression analyses were used to calculate individual effects of multiple variables on incisional hernia incidence. Parameters with p-values less than 0.1 in univariate analyses were included in multivariate analyses to identify independent risk factors. p-values less than 0.05 were considered significant [28].

## Results

A complete follow-up of 753 patients was performed. The mean follow-up was 60.2 months (median 62, range 0-100). Of the 753 patients, 342 (45.4%) underwent cholecystectomy and the remaining 411 (51.6 per cent) underwent inguinal hernia repair. The study collective consisted of 405 women (53.8%) and 348 men (46.2%). The mean age at the date of surgery was 40 years (median 36 years). Details regarding the follow-up are displayed in Table 1. Relationship between the incidence of umbilical hernia and the length of follow-up time is shown in Table 2. Mean BMI of the study group was 21.9 kg/m2 (median 24.4, range 17.2–45.0). An overview of the study collective is shown in Table 3.

During follow-up, 10 patients developed umbilical hernias, of which eight (80 per cent) underwent cholecystectomy and two (20 per cent) underwent inguinal hernia repair. The mean BMI of patients who developed umbilical hernia was 33.2 kg/m^2^ (median 34.1, range 28.1–46.2). The relationship between incisional hernia incidence and follow-up time is shown in the Table 2 below.

**Table 1:** Follow-up

Follow-up time(months)	60.2 ± 4.2
Pain or discomfort at the scar	1.50 ± 1.5
Umbilical swelling	5.6%(*n* = 42)
Incisional hernia	1.6%(*n* = 10)
Overall satisfaction	98.4%(*n* = 741)

Values as numbers and percentages or in means ± standard deviation

**Table 2:** Relationship between the incidence of umbilical hernia and the length of follow-up time

Follow-up time(months)	12	24	36	48	60
Umbilical hernia	2(20%)	5(50%)	7(70%)	10(100%)	10(100%)

**Table 3:** Study collective characteristics(*n* = 753)

Sex	
Female	405(53.8%)
Male	348(46.2%)
Age(years)	40.26 ± 23.4
Height(cm)	166 ± 17
BMI(kg/m^2^)	21.9 ± 5.2
ASA	
1	642(85.3%)
2	101(13.4%)
3	8(1%)
4	2(0.3%)
Procedure	
cholecystectomy	342(45.4%)
Inguinal hernia repair	411(54.6%)
Comorbidity	
Hypertension	126(16.7%)
Diabetes	89(11.8%)
Earlier abdominal surgery	147(19.5%)
Conversion to multiport laparoscopy	1(0.13%)
Duration of the operation(min)	26.40 ± 15.2
Length of hospital stay(days)	2 ± 1
Pre-existing umbilical hernia	26(3.5%)
Intraoperative complication	1(0.1%)
Postoperative complication	12(1.6%)

Values as numbers and percentages or in means ± standard deviation ASA American Society of Anaesthesiologists, BMI Body Mass Index

**Table 4:** Univariate analysis of factors associated with umbilical hernia occurrence in patients undergoing SILS

	*n*	Event	OR	95%CI	*P* value
Sex					
Female	405	7	Reference		
Male	348	3	1.42	0.52—3.86	0.862
Age(years)					
<30	312	2	Reference		
≥ 30	441	8	1.76	0.92—7.16	0.962
BMI					
<30	592	1	Reference		
≥ 30	161	9	18.56	5.76—86.42	0.003
Height(cm)					
<170	412	6	Reference		
≥ 170	341	4	0.76	0.58—10.26	0.626
Weight(kg)					
<75	451	4	Reference		
≥ 75	302	6	1.58	0.96—15.42	0.512
Comorbidities					
Diabetes	128	4	2.42	1.86—20.42	0.496
Hypertension	265	2	1.96	0.72—12.64	0.924
Procedure					
Cholecystectomy	342	7	3.42	0.82—18.44	0.632
Inguinal hernia repair	411	3	2.38	0.42—22.48	0.422
Pre-existing umbilical hernia					
No	727	4	Reference		
yes	26	6	16.32	4.26—61.68	0.002
Time of surgery(min)					
<30	624	5	Reference		
≥ 30	129	5	1.28	0.72—9.22	0.566
Earlier abdominal surgery					
yes	356	5	Reference		
no	397	5	0.78	0.56—7.80	0.824

ASA American Society of Anesthesiologists, BMI body mass index, CI confidence interval, OR odds ratio

**Table 5:** Multifactorial analysis of the incidence of umbilical hernia in patients undergoing SILS

	OR	95%CI	*P* value
BMI	9.56	6.34—18.42	0.068
Pre-existing umbilical hernia	42.22	8.12—76.24	0.002

Based on our statistics, it can be noted that only one patient out of 753 cases was converted to conventional multi-port surgery during the procedure, with a single port completion rate of 99.9%. The complication rate of intraoperative bleeding was 0.1%. A total of 10 (1.6%) patients developed postoperative umbilical hernias during the follow-up period, of which 7 (70%) underwent cholecystectomy and 3 (30%) underwent inguinal hernia repair. The mean BMI of patients who developed umbilical hernia was 33.2 kg/m^2^ (median 34.1, range 28.1–46.2). Table 3 shows that complete detection of umbilical hernia takes at least 48 months. The univariate analysis related to the incidence of umbilical hernia is detailed in Table 4, which shows that obese patients (OR = 18.56, 95% CI 5.76–86.42, *P* = 0.003) and patients with pre-existing umbilical hernia (16.32, 95% CI 4.26–61.68, *P* = 0.002) were significantly associated with the occurrence of umbilical hernia. In addition to this, gender, age, type of surgery, duration of surgery, and underlying disease were also studied and none of these factors were statistically significant. In addition to this, two factors, BMI and Yoshimura umbilical hernia, were analysed in a multifactorial analysis and the results are shown in Table 5. BMI and pre-existing umbilical hernia were statistically significantly associated with high incidence of postoperative umbilical hernia. (OR = 9.56, 95%CI 6.34–18.42, *P* = 0.068 and OR = 42.22, 95%CI 8.12–76.24, *P* = 0.002).

## Discussion

Since its inception, the SILS technique has been receiving a lot of attention from minimally invasive surgical teams [29]. The lead surgeon in our team has been performing SILS routinely for almost two decades, so he is very skilled in the technique and is also very interested in its use and evaluation in clinical practice. However, from the recent relevant literature, we found that some experts have questioned the benefits of SILS for patients. The high incisional hernia rate after SILS has also been reported in the literature [30], and our team is puzzled by the fact that we rarely see patients with umbilical hernias during the daily surgical procedures and postoperative reviews. With this in mind we retrospectively analysed the clinical data of the patients who attended the clinic in the last five years or so and analysed our findings and techniques.

Based on the statistical analysis, we can find that, in our team, the incidence of postoperative umbilical hernia is only 1.6%, although this may be further reduced due to the return bias and the reluctance of the healed patients to undergo long-term follow-up [18]. Prof Comajuncosas et al. published a prospective study on incisional hernias, which reported an alarming incidence of 25.9% [9], and I feel that the reason for such a discrepancy is that the inflammatory response was not well managed preoperatively. And their team chose a supraumbilical incision, which leaves a cavity during postoperative suturing due to visual blindness.

The incidence of postoperative umbilical hernia was strongly correlated with BMI and the presence of a previous umbilical hernia [31]. We found that compared to Western populations, we Asians may have a more balanced diet, with less intake of high-sugar and high-calorie diets, and thanks to the habit of light eating, the BMI of Asian populations is generally lower, and therefore the number of our patients with umbilical hernias due to larger BMIs would be much lower during the course of a large number of statistical follow-up visits [32].

In addition, although the statistics do not reflect whether infection increases the incidence of umbilical hernia, close preoperative antimicrobial and anti-inflammatory treatment, as well as careful postoperative care, will greatly reduce the incidence of postoperative umbilical infection and even umbilical hernia. At our medical center, all patients undergoing single-incision laparoscopic surgery (SILS) are typically discharged on postoperative day 1 to 2. For patients without specific drug allergies, a standardized protocol of intravenous cephalosporin antibiotics is administered for 1 day, followed by discharge with a 7-day oral cephalosporin antibiotic regimen. Patients experiencing intolerable pain receive appropriately titrated analgesics or are prescribed pinaverium bromide for outpatient use. The clinical efficacy of these interventions is currently under investigation in ongoing studies.

We have noticed that many operations for SILS are not performed with an incision at the location of the navel, but take a supra-umbilical or infra-umbilical position for the operation [9]. We believe that such a surgical approach does not result in cosmetic gain, and we usually take the navel, a natural orifice, for surgery, which allows adequate exposure of the umbilical ring and fascia, and is more conducive to surgical manipulation as well as postoperative gapless suturing. This is the only way we can truly perform scarless surgery.

Our team has been performing SILS routinely for more than 20 years [33], and the surgeons are very skilled in the surgical procedure and the layout of the instruments. As a result, we are able to operate in as little as 25 min or even less. This means less intraoperative trauma for the patient and fewer postoperative complications [30].

The present retrospective analysis of patient data over a period of about five years suggests that a longer follow-up period, with larger and more detailed clinical data, is needed to study the incidence of umbilical hernia after SILS. This study is limited by the fact that the attending surgeon routinely performs SILS surgery and does not count data from routine laparoscopic surgery, which is best done as a cohort study [34]. There may be some selection bias in this patient data, and healed patients may ignore this study, resulting in large actual results. Perhaps the presence of a smaller umbilical hernia postoperatively was not brought to the attention of the patient, resulting in a small result.

## Conclusion

SILS has recently been introduced as an evolution in minimally invasive surgery and is used for an ample variety of intra-abdominal operations, including gynecologic and urologic procedures, adrenal, colon, pancreas, liver, and small-bowel resections [35]. SILS is a well-established technique, it offers less trauma, fewer complications, and cosmetic advantages [24]. The incidence of postoperative umbilical or incisional hernias is not related to this technique, but depends on the level of the surgeons.

## Data Availability

In this study, all the original data were sourced from the medical record room of the Affiliated Hospital of Nantong University. The study has passed the review of the Ethics Review Committee of the Affiliated Hospital, with the review number: 2023-k117-01. Relevant information can be obtained on our Data Availability Statement page. Out of consideration for the protection of patients’ privacy information and the safety of the Asian ethnic group, we are willing to provide detailed materials and operation-related videos to interested readers. Readers who are interested are welcome to send an email to johnny052766@163.com to obtain the detailed data. The data that support the findings of this study are available from the Affiliated Hospital of Nantong University. However, there are restrictions on the availability of these data. These data were used under license for the current study and thus are not publicly accessible. Nevertheless, the data can be obtained from the authors upon a reasonable request and with the permission of the Affiliated Hospital of Nantong University.
